# Osteoprotegerin in Exosome-Like Vesicles from Human Cultured Tubular Cells and Urine

**DOI:** 10.1371/journal.pone.0072387

**Published:** 2013-08-23

**Authors:** Alberto Benito-Martin, Alvaro Conrado Ucero, Irene Zubiri, Maria Posada-Ayala, Beatriz Fernandez-Fernandez, Pablo Cannata-Ortiz, Maria Dolores Sanchez-Nino, Marta Ruiz-Ortega, Jesus Egido, Gloria Alvarez-Llamas, Alberto Ortiz

**Affiliations:** 1 Department of Nephrology, Instituto de Investigaciones Sanitarias-Fundación Jiménez Díaz - Universidad Autonoma de Madrid, Madrid, Spain; 2 Department of Immunology, Instituto de Investigaciones Sanitarias-Fundación Jiménez Díaz - Universidad Autonoma de Madrid, Madrid, Spain; 3 Department of Pathology, Instituto de Investigaciones Sanitarias-Fundación Jiménez Díaz - Universidad Autonoma de Madrid, Madrid, Spain; 4 Instituto de Investigacion Hospital Universitario La Paz, Madrid, Spain; 5 Instituto Reina Sofia de Investigacion Nefrologica, Madrid, Spain; University of Louisville, United States of America

## Abstract

Urinary exosomes have been proposed as potential diagnostic tools. TNF superfamily cytokines and receptors may be present in exosomes and are expressed by proximal tubular cells. We have now studied the expression of selected TNF superfamily proteins in exosome-like vesicles from cultured human proximal tubular cells and human urine and have identified additional proteins in these vesicles by LC-MS/MS proteomics. Human proximal tubular cells constitutively released exosome-like vesicles that did not contain the TNF superfamily cytokines TRAIL or TWEAK. However, exosome-like vesicles contained osteoprotegerin (OPG), a TNF receptor superfamily protein, as assessed by Western blot, ELISA or selected reaction monitoring by nLC-(QQQ)MS/MS. Twenty-one additional proteins were identified in tubular cell exosome-like vesicles, including one (vitamin D binding protein) that had not been previously reported in exosome-like vesicles. Twelve were extracellular matrix proteins, including the basement membrane proteins type IV collagen, nidogen-1, agrin and fibulin-1. Urine from chronic kidney disease patients contained a higher amount of exosomal protein and exosomal OPG than urine from healthy volunteers. Specifically OPG was increased in autosomal dominant polycystic kidney disease urinary exosome-like vesicles and expressed by cystic epithelium in vivo. In conclusion, OPG is present in exosome-like vesicles secreted by proximal tubular epithelial cells and isolated from Chronic Kidney Disease urine.

## Introduction

Exosomes are small-sized nanovesicles originated inside the cell traffic network and secreted through the fusion of multivesicular bodies with the cell membrane [1], [2]. Exosomes and other microvesicles are found in most human fluids, including human urine, and are secreted by a wide range of cell types [3], [4]. Urinary exosomes have obtained wide attention regarding their potential role as disease biomarkers [5],[6],[7]. However, very little is known about exosome secretion by kidney cells, the composition of kidney cell exosomes or their function.

Unraveling the function of exosomes holds promise to develop new therapeutic approaches to human disease. Potential biological functions of exosomes include antigen presentation and regulation of programmed cell death, angiogenesis, inflammation and coagulation [3], [8]. Exosomes may carry morphogens, chemo-attractant signals, miRNAs and mRNAs [9]. T cell exosomes contain members of the TNF superfamily of proapoptotic cytokines such as TRAIL, TNF and FasL and their presence in exosomes is key to death of T cell target cells [10], [11]. In this regard, TNF superfamily proteins are often more lethal if anchored to a membrane surface than in solution [12]. Furthermore, members of the TNF receptor superfamily may also be present in exosomes. Microvesicles containing TNF-R1 function as decoys for TNF signaling [13]. Tubular cells express functional TNF superfamily proapoptotic cytokines such as TNF, Fas ligand, TRAIL and TWEAK [14], [15], [16], [17], [18]. Emphasizing the potential importance of these cytokines for kidney pathophysiology, a human kidney transcriptomics approach disclosed that TRAIL and its decoy receptor osteoprotegerin (OPG/OCIF/TNFRSF11B) [19] were the apoptosis-related genes most highly expressed in diabetic nephropathy (DN), the most frequent form of chronic kidney disease (CKD) [16]. Immunohistochemistry disclosed that tubular cells were the main source of TRAIL in DN [16]. In culture, there was functional evidence for the expression of OPG by tubular cells [16]. OPG is a TNF receptor superfamily glycoprotein of 401 amino acids, frequently attached to various proteoglycans [20] ^,^ [21]. OPG was initially described as a decoy receptor for receptor activator of NFκB ligand (RANKL) that regulates osteoclastogenesis [20]. Serum levels of OPG are increased in CKD patients and have been associated with vascular calcification [22].

We have now addressed the composition of tubular cell-derived exosome-like vesicles by two complementary approaches. First, we explored the presence in human proximal tubular cell-derived exosome-like vesicles of selected TNF superfamily proteins and receptors. Specifically we focused on those most highly expressed in DN, TRAIL and OPG. In addition, we employed a proteomics approach to identify additional components of tubular epithelial cell exosome-like vesicles that might shed light onto their function. We did not find TRAIL in proximal tubular cell exosome-like vesicles. Surprisingly, OPG was identified as a tubular cell exosomal protein by a variety of techniques. We also show for the first time that exosomal OPG is increased in urinary exosome-like vesicles from CKD patients.

## Materials and Methods

### Cell Culture

HK-2 human proximal tubular epithelial cells (ATCC, Rockville, MD) were grown on RPMI 1640 (GIBCO, Grand Island, NY), 10% heat-inactivated fetal bovine serum, 2 mM glutamine, 100 U/mL penicillin, 100 µg/mL streptomycin, 5 µg/mL insulin, 5 µg/mL transferrin, 5 ng/mL sodium selenite, and 5 ng/mL hydrocortisone in 5% CO_2_ at 37°C as previously described [23]. For experiments cells were rested in serum-free media for 24 h prior to sample collection.

### Clinical Characteristics of Patients

Second morning void urine samples were obtained from human healthy volunteers or stable CKD patients who donated urine samples. All the study “OPG in exosome-like vesicles from human cultured tubular cells and urine”, and the human samples used, has been approved by the IIS-Fundacion Jimenez Diaz Biobank Local Ethics Biobank. Informed consent were signed by all the patients involved in the study, and all clinical investigation were conducted according to the principles expressed in the Declaration of Helsinki. Clinical characteristics are summarized in **Table 1**. The eGFR was estimated from serum creatinine using the Modification of Diet in Renal Disease (MDRD) Study Group equation: eGFR (ml/min/1.73 m2) = 186×(SCr)−1.154×(age in years)−0.203×(0.742, if patient is female)×(1.212, if patient ethnicity is black) [24]. Healthy controls were 3 males and one female, 61±10-year-old, non-diabetics, with serum creatinine <1.2 mg/dl and albuminuria <30 mg/g creatinine. A cocktail of protease inhibitors (2 mM AEBSF, 0.3 µM aprotinin, 130 µM bestatin, 1 mM EDTA, 14 µM E-64, 1 µM leupeptin, Sigma-Aldrich) were added to 100 ml urine and samples were frozen at −80°C until microvesicle extraction.

**Table 1:** Clinical characteristics.

Gender	Age (years)	DM	Cause of CKD	sCr (mg/dl)	eGFR (ml/min)	UProt (mg/dl)
M	46	Yes	DN	3.8	18	32
M	83	Yes	DN	2.9	22	130
M	79	Yes	DN	1.6	13	296
M	69	No	GN	6	5	185
F	50	No	CAKUT	3.5	15	38
M	55	No	ADPKD	4.6	14	86
M	68	No	ADPKD	6.7	9	196
M	29	No	ADPKD	1.2	81	13
M	65	No	ADPKD	2.4	29	6
F	74	No	ADPKD	3.2	15	47
M	53	No	ADPKD	6.2	8	47
M	65	No	ADPKD	1.4	54	4
F	62	No	ADPKD	2.3	23	4
F	76	No	ADPKD	5.4	8	17
Summary data	62±15			3.66±1.86	22±21	79±90

Summary data expressed as mean±SD.

sCr: serum creatinine, eGFR: estimated glomerular filtration rate, UProt: urinary protein.

### Microvesicle Isolation

Microvesicles were obtained by serial ultracentrifugation at 4°C. In each centrifugation the pellet was discarded and the supernatant used in the next step. The first steps eliminate dead cells and large cell debris [25]. For cell culture supernatants, we normalized the values to 1 ml of culture medium conditioned per million cells. The yield was 1251±349.95 ng of exosomal protein/million cells. The isolation starts with a 300×g (cells and cell debris removal) centrifugation for 10 minutes; 1,100×g for 10 minutes twice; 6,000×g for 10 minutes; 17,000×g for 30 minutes and 200,000×g for 70 minutes. This last step precipitates small particles corresponding to exosome-like vesicles secreted into the culture medium. This pellet was washed with a large volume of PBS to avoid contamination with soluble proteins and then centrifuged again for 70 minutes at 200,000×g. The final pellet obtained was resuspended in various solvents (PBS, lysis buffer), depending on the purpose of the extraction. For urine samples we followed a modified protocol, optimized for urine. After thawing the 50–100 ml samples, an aliquot was removed and whole urine was centrifuged at 17,000×g for 30 minutes. The pellet was resuspended in isolation buffer (200 mM sucrose, 10 mM triethanolamine) and dithiothreitol (DTT) was added (200 mg/ml) to disrupt Tamm-Horsfall protein network that entraps exosome-like vesicles, thus increasing isolation efficiency [4], [26]. After adding DTT, the solution was centrifuged again at 17,000×g for 10 minutes, the pellet containing precipitated Tamm-Horsfall protein was discarded and the supernatant containing exosome-like vesicles was mixed with the first centrifugation supernatant kept at 4°C. The newly reconstituted samples were centrifuged at 200,000×g for 70 minutes at 4°C. The pellet obtained was resuspended in PBS or lysis buffer as required. Exosomal protein was assessed by the Bradford assay. All centrifugations were performed following described standard procedures [27], using a 70 Ti fixed angle rotor with 26 ml polycarbonate tubes. Conversion from rpms to RFCs (Relative Centrifugal Force; ǵs) were made automatically by the centrifuge.

### ELISA

OPG was quantified by ELISA (Biomedica Gruppe) following the manufacturer instructions. Proteins from the following origins were assayed: tubular epithelial cells, whole human urine, tubular cell culture supernatant, tubular cell derived exosome-like vesicle and urinary exosome-like vesicles. Optical density (OD) was read in all wells on a plate reader using 450 nm wavelength (correction wavelength 630 nm). We construct the standard curve from the OD values of an OPG standard with a range 0 to 20 pmol/l and obtained the sample concentration from this standard curve. The detection limit of the assay is 0.07 pmol/l.

### Transmission Electron Microscopy

The pellet containing exosome-like vesicles was resuspended in 2% paraformaldehyde/PBS, and 5 µl were plated on Formvar/carbon-coated grids (Ted Pella Inc. CA, USA). After adsorption, grids were washed several times in PBS and transferred to 1.5% glutaraldehyde for 5 minutes. After another series of washes, grids were transferred to a uranyl-oxalate solution for 5 minutes and to methylcellulose-uranyl-oxalate for 10 minutes. Grids were dried and observed in a transmission electron microscope at 80 kV.

### Flow Cytometry

Because of their small size, exosomes can only be analyzed in a flow cytometer after linkage to larger particles of known size. Exosome-like vesicles were adsorbed to solid 3.9 mm latex microspheres composed of aldehyde-free sulfate surfactants (Interfacial Dynamics, USA). Microspheres/exosomes were incubated with anti-CD63-PE antibody (Becton-Dickinson) and analyzed on a FACScalibur flow cytometer (Becton-Dickinson).

### Western Blot

Western blots were performed as previously described [28]. Membranes were incubated overnight at 4°C with anti-OPG antibody (1∶500, Acris Antibodies GmbH, Germany), anti-TRAIL (1∶1,000, R&D systems), anti-ALIX (1∶1000; Santa Cruz Biotechnology, CA, USA), anti-TSG101 (1∶1000; AbCam, Boston, MA, USA), anti-CD63 (1∶2000; Millipore, MA, USA), anti-Calnexin (Calbiochem), anti-Tweak (1∶500, Santa Cruz Biotechnology), anti-Fibronectin (1∶1000, Millipore, MA, USA), anti-C3 (kind gift from Dr. Pastor-Vargas) and anti-TGFβ-iH3 (1∶1000, Santa Cruz Biotechnology, CA, USA) followed by incubation with horseradish peroxidase-conjugated secondary antibody (1∶2,000, Amersham, Aylesbury, UK). The use of a different anti-OPG antibody (Santa Cruz) yielded results similar to those shown in the figures. Blots were developed with the enhanced chemiluminescence method (ECL) following the manufacturer’s instructions (Amersham). All assays were performed under reducing conditions (adding β-mercaptoethanol as reducing agent into the loading buffer) except for CD63. Autoradiographs were scanned using the GS-800 Calibrated Densitometer (Quantity One, Bio-Rad).

### Sample Preparation for LC-MS/MS

Exosomal protein extract (50 µg) from tubular epithelial cell supernantants was concentrated and desalted. In a first attempt electrophoresis was performed until the sample passed through the stacking gel and reached the running gel. At that moment, electrophoresis was stopped and the gel was stained using colloidal Coomassie (Fermentas). The piece of stained gel in which the sample was concentrated was excised and digested. In this way, all proteins were concentrated in a unique band, eliminating sample contaminants [29]. To further increase the number of identified proteins, full electrophoresis was additionally performed and the stained gel pieces were excised. Digestion was performed according to Schevchenko *et al* [30] with minor modifications: the gel piece was incubated with 10 mM DTT in 50 mM ammonium bicarbonate (99% purity; Scharlau) for 30 min at 56°C and after reduction, alkylated with 55 mM iodoacetamide (Sigma Aldrich) in 50 mM ammonium bicarbonate for 20 min at RT. Gel plugs were washed with 50 mM ammonium bicarbonate in 50% methanol (gradient, HPLC grade, Scharlau), rinsed in acetonitrile (gradient, HPLC grade, Scharlau) and dried in a Speedvac. Dry gel pieces were then embedded in sequencing grade modified porcine trypsin (Promega, Madison, WI, USA) at a final concentration of 20 ng/µL in 20 mM ammonium bicarbonate. After digestion at 37°C overnight, peptides were extracted with 60% acetonitrile (ACN) in 0.1% formic acid (99.5% purity; Sigma Aldrich) and the sample were resuspended in 98% water with 2% formic acid (FA) and 2% ACN.

### LC-MS/MS and Database Searching

The LC/MSMS system consisted of a TEMPO nano LC system (Applied Biosystems) combined with a nano LC Autosampler. LC-MS/MS analysis was performed on an AB/MDS Sciex 4000 Q TRAP System with NanoSprayII Source (Applied Biosystems). Two replicate injections (4.5 µL) were made for each sample using mobile phase A (2% ACN/98% water, 0.1% FA) with a flow rate of 10 µL/min for 5 min. Peptides were loaded onto a µ-Precolumn Cartridge (Acclaim Pep Map 100 C18, 5 µm, 100 Å; 300 µm i.d. X 5 mm, LC Packings) to preconcentrate and desalt samples. RPLC was achieved on a C18 column (Onyx Monolithic C18 150×0.1 mm, Phenomenex) using mobile phase A (2% ACN/98% water, 0.1% FA) and mobile phase B (98% ACN/2% water, 0.1% FA). Peptides were eluted at a flow rate of 300 nL/min by the following gradient: initial conditions 5% B, increased to 40% B over 40 min, 40 to 95% B for 1 min and then 95% B for 4 min, returning next to initial conditions (5% B) in 2 min and maintaining them for 14 more minutes.

The TEMPO nano LC system and 4000 QTRAP were both controlled by Analyst Software v.1.5.1. Analyst software creates wiff format files including all the spectra data. Wiff files were batch-processed by ProteinPilot™ Software 2.0.1 (Applied Biosystems/MDS Sciex) which automatically generated peak lists that were searched against the Swissprot database version 2011_01 using MASCOT version 2.2 (Matrix Science) [31], with 0.8 Da precursor and MS/MS fragment tolerance, allowing 1 missed cleavage and setting carbamidomethyl cysteines and methionine oxidation as modifications.

All the MS and MS/MS data were achieved in positive ion mode with an ion spray voltage of 2800 V, a declustering potential of 80 V and a nanoflow interface temperature of 150°C. Nitrogen was applied as both curtain and collision gas, setting source gas 1 and curtain gas to 20 psi. An Information Dependent Acquisition (IDA) method was programmed, with a full scan Enhanced MS (EMS) experiment at 4000 amu/s for ion profiling, followed by an enhanced resolution (ER) MS experiment at 250 amu/s. ER experiment allowed charge state recognition, further used by the IDA criteria to select precursor ions and to estimate the collision energy to fragment them. This IDA criteria was set to select the 8 doubled, tripled or quadrupled charged most intense ions from 400–1200 m/z that exceed 100,000 counts for fragmentation in the LINAC collision cell, excluding isotopes within a 4.0 amu window and with a mass tolerance of 1000,000 mmu. These 8 ions were submitted to 8 independent Enhanced Product Ion (EPI) MS/MS experiments at 4,000 amu/s with Dynamic Fill Time (DFT). The total number of MS and MS/MS experiments per cycle was 10 (1 EMS, 1 ER and 8 EPI), resulting in a total cycle time of 5.0058 s.

### OPG Analysis by Selected Reaction Monitoring (SRM) in a QQQ-LC/MS

SRM was performed to specifically identify OPG in exosome-like vesicles. Exosome-like vesicles were lysed in 7 M urea, 2 M thiourea and 4% Chaps and proteins were precipitated in acetone and solved in 8 M urea, reduced with 10 mM DTT (Sigma Aldrich) for 30 min at 37°C and alkylated for 20 min at room temperature with 55 mM iodoacetamide (Sigma Aldrich). Proteins were then digested in 50 mM ammonium bicarbonate (pH 8.5), with sequencing grade modified bovine trypsin (Merck) at a final concentration of 1∶50 (trypsin:protein). After overnight digestion at 37°C, 1 µl trifluoroacetic acid (Merck) was added to stop the reaction and tryptic peptide solutions were cleaned with C18 spin columns (Protea Biosciences) according to the manufacturer’s instructions. Tryptic digests were dried in a Speedvac and resuspended in mobile phase A (5% acetonitrile (Merck), 0.1% formic acid (Fluka)). Samples were analyzed in Selected Reaction Monitoring (SRM) mode using a 6460 Triple Quadrupole LC/MS/MS on-line connected to an nLC-ChipCube interface (Agilent Technologies) and 1200 Series LC Modules (Agilent Technologies) provided with a pre-cooled nLC autosampler.

The following is the FASTA sequence for OPG, where the 4 measured peptides are in bold and underlined:

MNNLLCCALVFLDISIKWTTQETFPPK**YLHYDEETSHQLLCDKCPPGTYLK**QHCTAKWKT

VCAPCPDHYYTDSWHTSDECLYCSPVCKELQYVKQECNRTHNRVCECKEGRYLEIEFCLK

HR**SCPPGFGVVQAGTPER**NTVCKRCPDGFFSNETSSKAPCRKHTNCSVFGLLLTQK**GNAT**

**HDNICSGNSESTQK**CGIDVTLCEEAFFRFAVPTKFTPNWLSVLVDNLPGTKVNAESVERI

KRQHSSQEQTFQLLKLWKHQNKDQDIVKKIIQDIDLCENSVQRHIGHANLTFEQLRSLME

SLPGKKVGAEDIEKTIKACKPSDQILKLLSLWRIKNGDQDTLKGLMHALKHSKTYHFPKT

VTQSLKKTIRFLHSFTMYKLYQKLFLEMIGNQVQSVKISCL

Peptide separation was carried out onto a ProtID chip with 43×0.075-mm analytical column and 40 nL enrichment column (Agilent Technologies). Samples were injected at 4 µL/min and separation took place at 0.8 µL/min in a continuous acetonitrile gradient: 5–30% B at 0.5 min, 30–99% B at 1 min and 99% B at 3.5 min (phase B: 100% acetonitrile, 0.1% formic acid). Mass Hunter Software (v4.0) (Agilent Technologies) controlled the system. The mass spectrometer was operated in positive ion mode with capillary voltage of 1950 V, 325°C source gas temperature and 5 L/min source gas flow. Fragmentor was set to 130 V, dwell time to 250 ms, delta EMV to 200 V and collision energy was optimized for each SRM transition. SRM methodology allows specific detection of OPG proteotypic peptides. Theoretical SRM transitions were designed using Skyline (v.1.1.0.2905) [32] and manually inspected. OPG specificity was confirmed by protein blast and prototypic peptides were selected for SRM analysis.

### OPG Immunohistochemistry

The site of OPG protein expression in the kidney cortex was localized by immunohistochemistry in 4 nephrectomy specimens from non-diabetic patients obtained from the IIS-Fundacion Jimenez Diaz biobank: A sample with preserved kidney histology from a 76-year-old male suffering from kidney cancer with serum creatinine 0.9 mg/dl and estimated glomerular filtration rate (eGFR) >60 ml/min; another kidney from a 74-year-old male had histological features of CKD secondary to ureteral obstruction by urothelial cancer, with serum creatinine 1.6 mg/dl and eGFR 45 ml/min. In addition 10 renal cysts from 2 autosomal dominant polycystic kidney disease (ADPKD) patients on dialysis, male and female, 54- and 62-year-old were studied. Paraffin-embedded sections 5 µm thick were processed as described [23] and antigen retrieval was performed at pH 9 (Dako). The primary antibody was polyclonal rabbit anti-human OPG (1∶500; Acris) and secondary antibody was HRP-conjugated. Sections were counterstained with Carazzìs hematoxylin. Negative controls included incubation with a non-specific immunoglobulin of the same isotype as the primary antibody.

### Database Mining

In order to put the results in context and further define the potential clinical and biological implications the sub-set of proteins compiled in Table 2 was additionally analyzed by the following databases, Kidney & Urinary Pathway Knowledge Base [33], Nephromine [34] and STRING 9.0 software [35], consisting of a database of known and predicted protein interactions, including direct (physical) and indirect (functional) associations derived from genomics, high-throughput experiments, coexpression and previous knowledge. The highest confidence (0.900) filter was applied.

**Table 2:** Proteins identified in tubular epithelial cell-derived exosomes by SDS-PAGE and LC-MS/MS analysis.

Gene	Protein	UniProtKB/Swiss-Prot	Conf. (%)	Exocarta
AGRN	Agrin	Junction formation and maintenance.Basement membrane. ECM	99	Y
ACTBL2	Beta-actin-like protein 2	Cell motility	99	Y
C3	Complement C3	Activation of the complement system.Innate immunity	99	Y
CHRDL1	Chordin-like protein 1	Antagonizes the function of BMP4(Bone morphogenic protein)	99	Y
COL4A1	Collagen alpha-1 (IV) chain	Structural component of glomerularbasement membranes. ECM	99	Y
COL4A2	Collagen alpha-2(IV) chain	Basement membrane. ECM	99	Y
LGALS3BP	Galectin-3-binding protein	Promotes integrin-mediated cell adhesion. ECM	99	Y
FBLN1	Fibulin-1	Role in cell adhesion and migration.Basement membrane. ECM	99	Y
FN1	Fibronectin	Involved in cell adhesion and migration. ECM	99	Y
THBS1	Thrombospondin-1	Cell-to-cell and cell-to-matrix interactions.Binds heparin. ECM	99	Y
TINAGL1	Tubulointerstitial nephritis antigen-like	Non-catalytic peptidase C1 family protein	99	Y
TGFBI	TGF-β-induced protein ig-h3	Cell-collagen interactions. ECM	99	Y
CYR61	Protein CYR61	Proliferation, chemotaxis,angiogenesis and cell adhesion. ECM	98	Y
HSPA4L	Heat shock 70 kDa protein 4L	Chaperone activity	98	Y
PKM	Pyruvate kinase, muscle	Glycolytic enzyme involved in ATP generation	98	Y
SRBD1	S1 RNA-binding domain-containing protein 1	Nucleobase-containing compound. RNA binding.	97	Y
VCP	Valosin containing protein	Involved in the formation ofthe transitional endoplasmic reticulum	96	Y
PXDN	Peroxidasin homolog	Peroxidase activity. ECM	95	Y
GC	Vitamin D-binding protein	Multifunctional protein found in biological fluids	92	N
LAMA5	Laminin subunit alpha-5	Cell migration. Basement membrane. ECM	92	Y
NID1	Nidogen-1	Basement membrane. ECM	92	Y

Exocarta: Presence (Y) or absence (N) in the exosomal database Exocarta, accessed December 28, 2012. [37]. In addition several keratins were identified (Table S1).

Conf.: confidence of the identification. ECM: extracellular matrix.

### Statistical Analysis

Results are expressed as mean ± standard error of mean. Differences between groups were assessed by the non-parametric test Mann-Whitney test and p<0.05 was considered significant. All the analysis was conducted using the SPSS statistical software (version 11.0).

## Results

### Proximal Tubular Epithelial Cells Release Microvesicles with Exosomal Features

**Figure 1:**
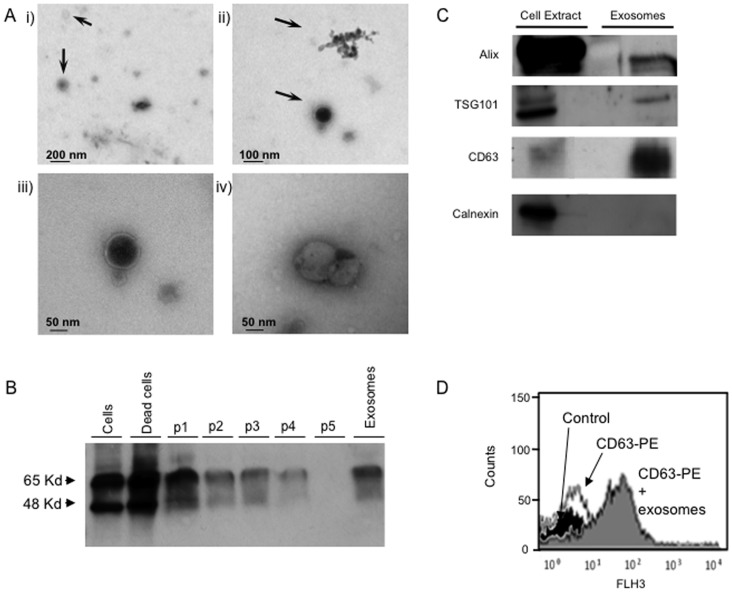
Characterization of exosomal-like vesicles isolated by serial centrifugation from cultured human proximal tubular epithelial cells. Microvesicles from tubular cell conditioned serum-free media present exosomal features. **A)** Transmission electron microscopy shows vesicles have a diameter 50–100 nm, consistent with exosomes. i) Scale bar = 200 nm, ii) Scale bar = 100 nm, iii & iv) Scale bar = 50 nm. Arrows point to exosomes. **B)** Representative Western blot for the exosome marker CD63. Each lane contains 20 µg protein obtained from the pellet of the following sequential centrifugation steps: P1 1,100×g, P2 1,100×g, P3 6,000×g, P4 17,000×g, and P5 100,000×g supernatant. Exo: exosomes present in the 200,000×g pellet. **C)** Standard exosome markers were present in exosomes secreted by HK2 cells (Western blot). Each lane contains 6 µg of exosomal proteins. **D)** CD63 expression assessed by latex bead flow cytometry. Black peak: control beads. White peak: beads+PE–conjugated anti-CD63. Grey peak: beads+PE–conjugated anti-CD63+5 µg exosomes. FLH3: fluorescence intensity.

Human proximal tubular HK2 cells constitutively release microvesicles with exosomal features when cultured in serum-free medium for 24 hours (Figure 1). Microvesicles were isolated following a standard differential centrifugation exosome purification protocol. Electron microscopy of the pellet obtained in the last centrifugation showed that tubular epithelial cells secrete microvesicles into the culture media. These vesicles were 50–100 nm in diameter, consistent with previous descriptions of exosomes (Figure 1A). To further characterize the purified vesicles as exosomes, we studied common exosomal proteins using Western blot and flow cytometry (Figure 1B–D). Pellets from the different centrifugation steps were assayed by Western blot to assess the exosomal tetraspanin CD63 (Figure 1C). Cells and dead cells/debris express CD63. CD63 expression decreases through the isolation process and is enriched in the final exosome-like vesicle pellet. Microvesicles secreted by tubular cells were also positive for Alix and TSG101 but calnexin was absent, indicating absence of cytoplasmic contamination (Figure 1C). Flow cytometry confirmed the presence of CD63 in exosomal vesicles from proximal tubular cells (Figure 1D). These features indicate that human tubular epithelial cells secrete exosome-like vesicles.

### Tubular Epithelial Cell-derived Exosome-like Vesicles Contain OPG but not TRAIL nor TWEAK

**Figure 2:**
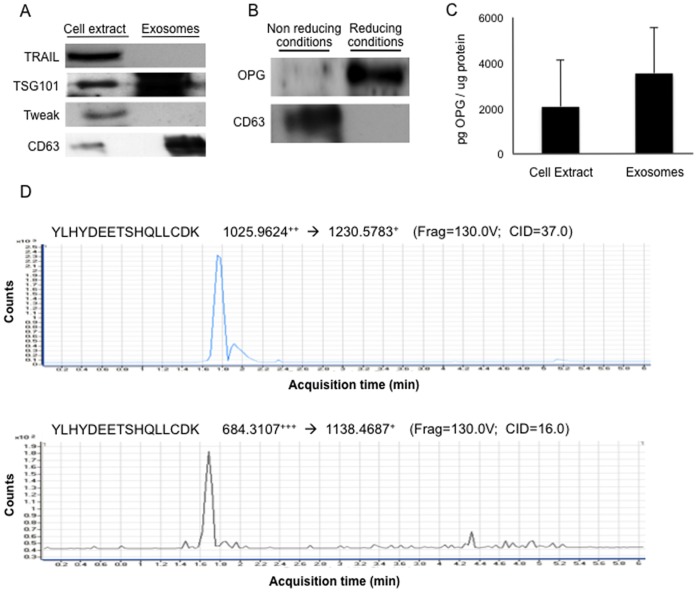
Proximal tubular cell exosomes contain OPG. **A)** Exosomes from HK-2 conditioned serum-free cell culture media. Representative Western blots of TRAIL, TWEAK and exosome markers. Each lane contains 5 µg exosomal protein. **B)** OPG is observed in HK-2-derived exosomes when reducing, denaturizing conditions are applied. Each lane contains 10 µg exosomal protein. **C)** OPG expression in HK-2-derived exosomes detected by ELISA. Results expressed as pg/µg of total protein. Mean+SEM of 3 independent experiments. **D)** OPG analysis by selected reaction monitoring (SRM) in a LC-(QQQ)-MS/MS showing two different transitions corresponding to the same precursor peptide which coelute in time. The mass and charge of the precursor and its fragments are shown. A single peptide (YLHYDEETSHQLL) and a single precursor were measured under two different charge state (1025.9624+2 and 684.3107+3), each of them with its own fragment (1230.5783+ and 1138.4687+, respectively), thus yielding two different peaks or transitions.

We have recently shown that cultured human tubular cells and tubular cells from human CKD biopsies express TRAIL, and that TRAIL promotes tubular cell death [16]. TRAIL is present in T cell-derived exosomes [36] and membrane bound-TRAIL is more lethal than soluble TRAIL. For these reasons we assessed the presence of TRAIL in exosomes secreted by human proximal tubular epithelial cells. However tubular epithelial cell-derived exosome-like vesicles did not contain TRAIL nor TWEAK, another TNF superfamily cytokine expressed by tubular cells (Figure 2A). Interestingly, anti-OPG antibodies identified a band of the expected size in Western blots run under reducing conditions (Figure 2B). A second, different anti-OPG antibody yielded similar results (not shown). The presence of OPG in exosomes secreted by tubular epithelial cells was confirmed using a specific ELISA assay for OPG (Figure 2C). We further confirmed the presence of OPG in tubular epithelial cell-derived exosome-like vesicles by selected reaction monitoring (SRM) in a QQQ-LC-MS/MS (Agilent Technologies). The aim of the SRM was solely to confirm specifically and unequivocally the presence of OPG in those samples, confirming WB data. In particular, two different transitions of the same precursor peptide could be detected co-eluting in time (Figure 2D). Three additional peptides were measured with several transitions per peptide (Figure S1).

### Additional Proteins Present in Proximal Tubular Epithelial Cell-derived Exosome-like Vesicles

**Figure 3:**
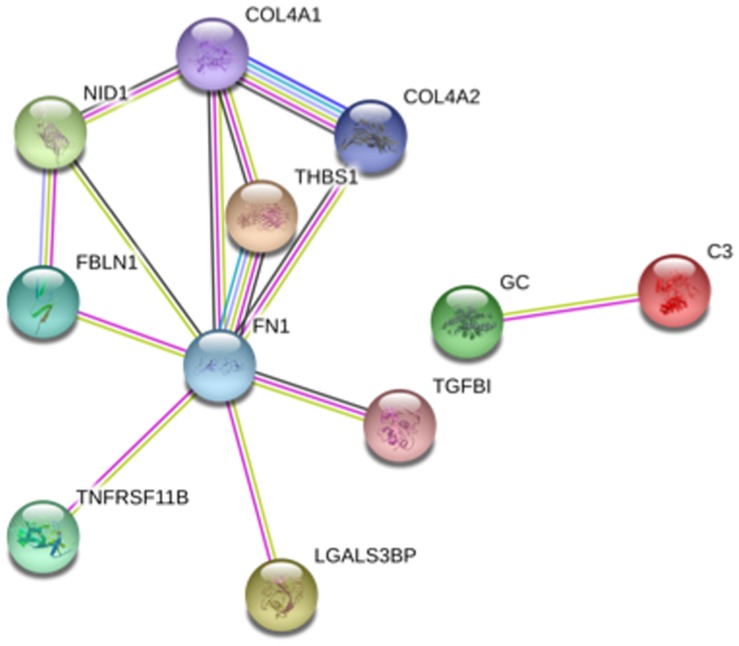
Relationships between OPG and proteins identified in tubular cell-derived exosomal-like vesicles by LC-MS/MS as evidenced by the STRING database. STRING map of predicted associations for the sub-set of proteins compiled in Table 2. The network nodes are proteins and the edges represent the predicted functional associations based on evidence which are represented by different color lines: green line, neighborhood in the genome; blue line, co-occurrence across genomes; purple line, experimental evidence; yellow line, text mining evidence; light blue line, database evidence; black line, co-expression evidence. The highest confidence filter was applied. NID1: Nidogen 1; COL4A1/2: Colagen 4 A1/2; THBS1: Thrombospondin-1; FBLN1: Fibulin 1, FN1: Fibronectin, TNFRSF11B: Osteoprotegerin; LGALS3BP: Galectin-3 Binding Protein, TGFBI: TGF-β-induced protein ig-h3, GC: Vitamin D-binding protein; C3: Complement C3.

Additional proteins present in exosome-like vesicles secreted by proximal tubular epithelial cells were identified by proteomic profiling. Exosomal protein extracts were concentrated and desalted by SDS-PAGE and identified by LC-MS/MS (Table S1). Twenty-one proteins were identified with a confidence ≥90%. Neither canonical exosomal markers nor OPG were identified by LC-MS/MS (Table 2). Most of the identified proteins had already been described in exosome-like vesicles as assessed by Exocarta [37]. However, this is the first time that vitamin D binding protein is described in exosome-like vesicles. Extracellular matrix proteins and, specifically, basement membrane proteins were overrepresented in tubular cell exosome-like vesicles. In this regard, nidogen-1 and agrin were only recently identified in exosome-like vesicles [37] and join type IV collagen, laminin, and fibulin-1 as basement membrane matrix proteins present in exosome-like vesicles. The presence of Fibronectin, C3 and TGFβ-ih3 was confirmed by Western blot (Figures S2 and S3). A search of the database STRING disclosed relationships between OPG and proteins identified in tubular cell-derived exosomal-like vesicles by LC-MS/MS (Figure 3).

### Exosome-like Vesicles are Present in Human Urine and Contain OPG

**Figure 4:**
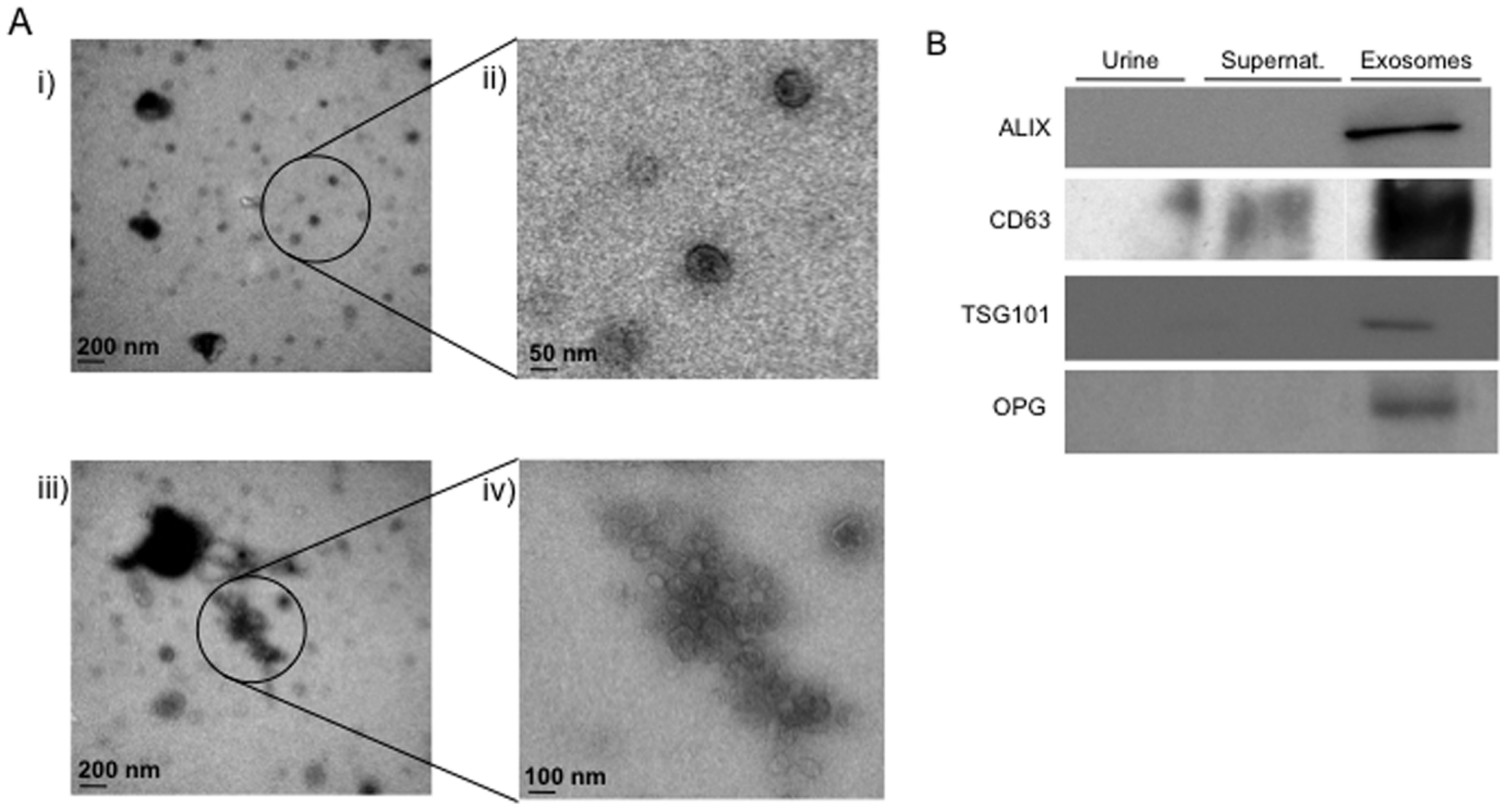
Exosomes from human urine contain OPG. **A)** Transmission electron microscopy representative images showing microvesicles purified from human urine. i) Scale bar = 200 nm; ii) Scale bar = 50 nm; iii) Scale bar = 200 nm; iv)Scale bar = 50 nm. ii&iv) show amplified areas from i&iii). **B)** Western blot. Representative images. Urine: whole urine collected from healthy donors. Supernat.: Urine supernatant from the last exosome isolation step. 5 µg protein were loaded per well.

The next step was to assess whether OPG was present in exosome-like vesicles from human urine samples. First we confirmed by electron microscopy that the isolation protocol applied to urine resulted in microvesicles with exosomal features in size and shape (Figure 4A). We confirmed the presence in these vesicles of the exosomal markers ALIX, CD63 and TSG101 (Figure 4B). As it was the case for exosome-like vesicles derived from human proximal tubular cells, human urine exosome-like vesicles contained OPG as assessed by Western blot (Figure 4B) and ELISA (3528±1994 pg OPG/µ g exosomal protein, not shown).

### OPG Expression is Increased in Exosome-like Vesicles from CKD Urine

**Figure 5:**
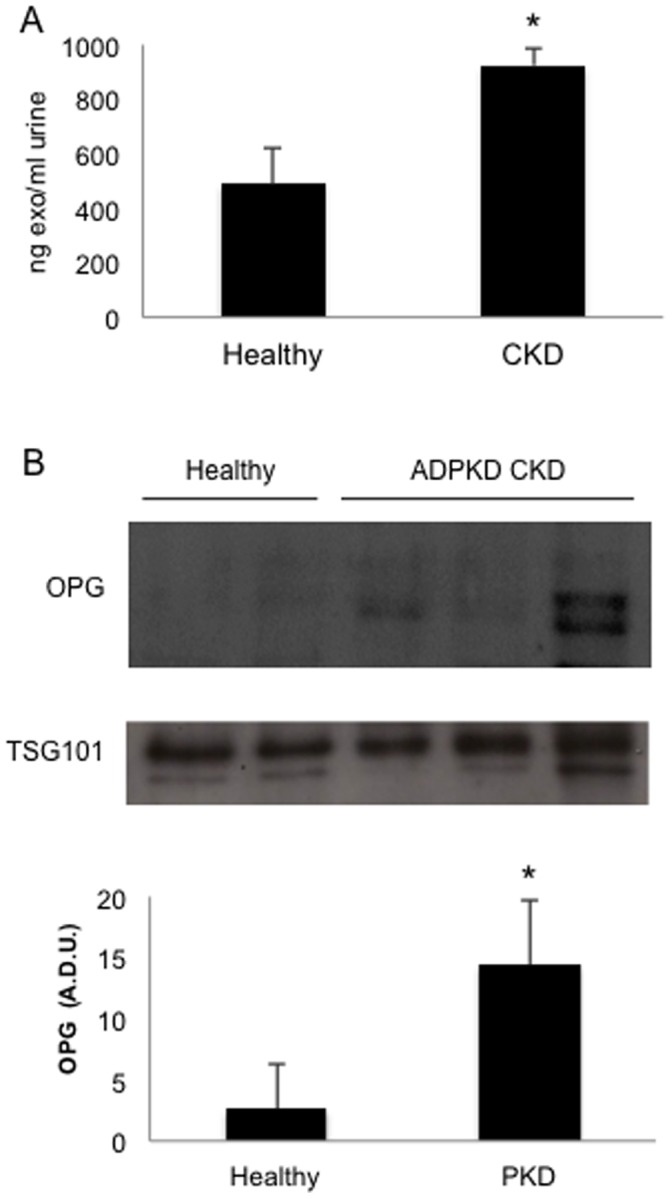
OPG in human urinary exosomes. **A)** Urinary exosomal protein content is higher in CKD patients (n = 14) than in healthy volunteers (n = 4), * p = 0.042. **B)** Representative Western blot. 5 µg urinary exosomal protein were loaded. All CKD samples in this blot correspond to ADPKD patients. **C)** Quantification of OPG levels as assessed by Western blot in ADPKD CKD patients (n = 9) and healthy volunteers (n = 4). Results presented as mean+SEM, * p = 0.0476. A.D.U.

An exploratory study in urine from healthy controls or CKD patients (Table 1) disclosed that urinary exosome-like vesicle protein content was higher in urine from CKD patients than in non-CKD controls (Figure 5A). OPG-containing exosome-like vesicles were detected in human urine samples, as assessed by Western blot, both in healthy volunteers and in CKD patients. Since in a preliminary analysis the highest OPG content corresponded to patients with ADPKD, further studies were performed in this population. OPG is higher in exosome-like vesicles obtained from ADPKD CKD patients than from healthy controls (Figure 5B). Increased levels were also found in diabetic nephropathy (n = 3, 5.87±0.28 Arbitrary Density Units, A.D.U.), IgA nephropathy (n = 1, 18.78 A.D.U) and CAKUT (Congenital anomalies of the kidney and urinary tract**)** (n = 1, 22.58 A.D.U)(Figure S4).

**Figure 6:**
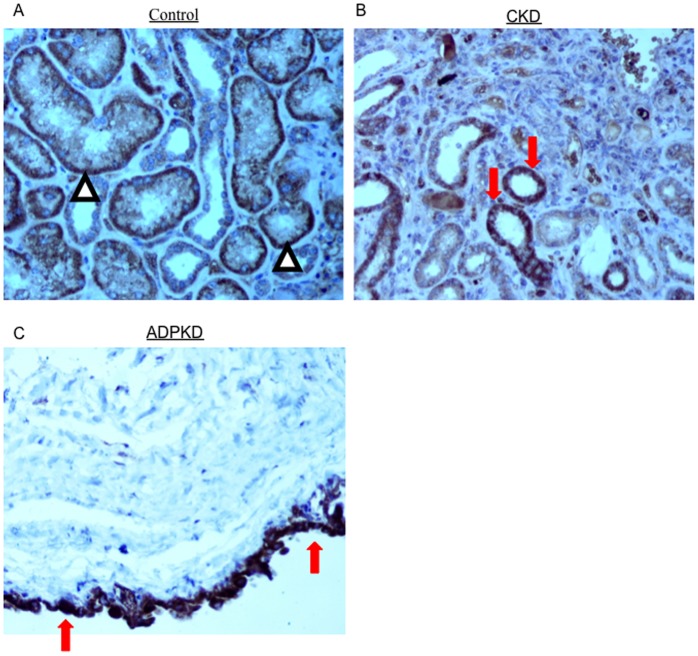
OPG immunohistochemistry in human kidneys. Control, CKD and autosomal dominant polycystic kidney disease kidneys samples were studied. For ADPKD a cortical cyst wall is shown. **A)** OPG mainly stained the basolateral aspect of proximal tubules in control kidneys (arrowheads). **B)** In CKD whole tubular cells are intensely stained in the cortex (arrows). **C)** A similar pattern of intense whole cell staining is observed in cyst epithelial lining (arrows). Original magnification ×200.

Increased OPG mRNA had been observed in the transcriptome from human DN biopsies [16]. However results from urinary exosome-like vesicles suggested that other nephropathies might be associated to increased kidney OPG. A search of the Nephromine database of published kidney transcriptome datasets disclosed that OPG mRNA was increased in the tubulointerstitium of a different DN data set (fold-change vs control: 2.4, p = 4.83E-4) as well as in IgA nephropathy (fold-change vs control: 1.7, p = 2.07E-4) [34], [38], [39], [40]. Furthermore renal cortex OPG gene expression correlated with chronicity index quartile (correlation: 0.497, p = 3.60E-5) in aging humans [34], [40]. OPG immunohistochemistry of human kidneys showed that OPG is mainly expressed in the basolateral aspect of proximal tubules in control kidneys (Figure 6). In CKD whole tubular cells are intensely stained in the cortex. A similar pattern of intense whole cell staining is observed in cyst epithelial lining in ADPKD.

## Discussion

Exosomes are secreted by most cell types and are also found in most biological fluids. Thus, tubular cells are expected to secrete exosomes and exosomes are found in the urine. However, there was no information on proximal tubular cell exosomes. We have shown for the first time that cultured human proximal tubular epithelial cells constitutively secrete exosome-like vesicles that contain the decoy receptor OPG as well as several other proteins, some not previously known to be exosomal proteins. The difference between various types of secreted microvesicles has generated a controversy in recent years and is not fully solved. Microvesicles isolated from tubular cell supernatants and from urine shared several exosomal characteristics, including their membrane-bound nature, the 50–100 nm size and the presence of exosomal markers such as CD63, TSG101, Calnexin and ALIX. However, increasing evidence has undermined the specificity of exosome markers and we cannot rule out that non-exosomal microvesicles are secreted by tubular cells and found in urine [41]. Although CD63 is much more abundant in late endosomes and lysosomes it is also expressed on cell surface [42] and nanotubes [43]. TSG101 is found in arrestin domain-containing protein 1-mediated microvesicles (ARMMs) [44] [45] and its role as a Endosomal Sorting Complexes Required for Transport (ESCRT) protein is far from being completely understood. The main functional family represented in proximal tubular cell exosome-like vesicles was the extracellular matrix, which points to a function of exosome-like vesicles in the modulation of cell-matrix interactions, normal matrix turnover or fibrosis. Furthermore, exosome-like vesicles containing OPG were found in urine from CKD patients and kidney OPG mRNA and protein is increased in diverse nephropathies and protein localized to tubular cells.

The function of exosomes is incompletely understood. Exosomes were first described as part of the cell protein recycling machinery and thought to function in the disposal of unwanted material [3], [46]. More recently their involvement in normal and abnormal intercellular communication has been emphasized. In this regard, exosomes contain mRNA and small RNAs, proteins and lipids [47]. One of the functions of exosomes is regulation of cell death. There is evidence supporting a role of cell death by apoptosis in the gradual loss of renal mass in CKD [15], [48]. T cell-derived exosomes are loaded with lethal TNF superfamily cytokines that increase their killing capacity both by providing simultaneously several lethal stimuli and by providing them on a surface rather than soluble [36], [49]. Conversely, TNF superfamily receptors in microvesicles may behave as decoys, dampening the response to TNF superfamily cytokines. Thus, microvesicles may contain TNF-R1 [13]. Our studies disclosed that TRAIL is not found in tubular cell exosome-like vesicles, unlike in exosomes from T cells. However, there is functional evidence that tubular cells are sensitive to TRAIL-induced death, at least under certain environmental conditions [16]. In this regard, TRAIL and OPG are the most overexpressed apoptosis-related genes in the most frequent cause of CKD, DN. OPG is a decoy receptor for TRAIL that is expressed by proximal tubular cells [19] [16], [50]. OPG expression by tubular cells protects them from TRAIL induced-apoptosis as evidenced by the increased rate of apoptosis when a specific antibody in tubular cells neutralized endogenous OPG [16]. The presence of OPG in tubular cell and urinary exosomes suggests that tubular cells may regulate different aspects of TRAIL and RANKL biology by secreting OPG in exosomes. We uncovered evidence in transcriptomics databases that suggests that increase kidney tubulointerstitial expression of OPG is found in diverse causes of CKD, including DN, glomerulonephritis and aging. OPG expression was localized to tubular cells in control and diseased kidneys by immunohistochemistry albeit the pattern of expression differed: luminal OPG expression was more prominent in CKD tubules and in cells lining ADPKD cysts. This location of OPG may contribute to OPG presence in urinary exosomes and, we speculate, even in kidney cyst fluid. The presence of OPG in exosomes may also be involved in the regulation of vascular calcification. Microvesicles are important in the calcification process and OPG has an anti-calcification role [20]. Fetuin, another factor that prevents vascular calcification, was recently reported to be present in kidney-derived exosomes [5]. The presence of OPG in circulating microvesicles should be explored since plasma OPG is associated with increased cardiovascular risk [51], [52].

While extracellular matrix proteins have long popped up in lists of exosomal proteins [37], [53], there is an insufficient understanding of the role of exosomes in the regulation of cell-matrix interactions, normal extracellular matrix deposition or fibrosis. Vesicle interaction with collagen types II and X is necessary for optimal bone calcification [54]. Exosomes derived from fibroblasts contain type I collagen [55]. Matrix metalloproteinases are also components of exosomes [56]. More recently exosomes derived from human umbilical cord mesenchymal stem cells were shown to regulate fibrosis in vivo [57]. In exosomes from proximal tubular epithelial cells a proteomics approach disclosed a striking presence of extracellular matrix proteins. Consistent with our understanding of tubular cell biology [58] several of these extracellular matrix proteins are known components of basement membranes, including two chains of type IV collagen (α1 and α2), laminin, nidogen-1, agrin and fibulin-1. However, there are no prior reports of such concentration of basement membrane proteins in exosomes from a particular cell type. This overrepresentation of extracellular matrix protein merits further functional studies under basal or stressed conditions in cultured cells as well as an exploration of their potential role as biomarkers in the clinical setting. Potential interactions between OPG and other proteins found in tubular cell-derived exosome-like vesicles were explored by database searches (KUPKB, STRING) and manual Pubmed searches. Complement 3 is involved in osteoclast formation in which OPG plays a role, but a direct interaction had not been described. OPG increases the arterial expression of TGF-β1 and promotes TGF-β1, fibronectin and collagen type IV expression in vascular smooth muscle cells [59]. Furthermore, TGF-β1 promoted the expression/release of endogenous OPG from vascular smooth muscle cells. OPG binds with high avidity to thrombospondin (TSP-1) [60]. In this regard, our studies do not address how is OPG linked to exosomes. One possibility is that OPG interacts with other membrane-bound proteins or proteoglycans, including thrombospondin-1. CYR61 has a role in proliferation and angiogenesis and is co-upregulated with OPG in an inflammatory osteocyte model [61]. As described in the UniProt KB database, some of the identified proteins are basement membrane related, including Col4A2, nidogen 1, laminin subunit alpha-5 and agrin. Agrin is preferentially expressed in kidney and lung [62].

The diagnostic potential of urinary exosomes is under evaluation [63]. Analysis of urinary exosomes may provide a non-invasive insight into events taking place in the kidney. Altered exosomal expression of aquaporins 1 and 2 has been reported in renal ischemia/reperfusion and antidiuretic hormone action [64]. Here we describe an increased amount of exosomal protein in the urine of CKD patients. This suggests that the CKD environment may regulate the secretion of exosomes, although the factors that may contribute to this observation are yet uncharacterized. Furthermore, we describe for the first time the presence of OPG in urinary exosomes, and its increased expression in patients with CKD. More specifically, we observed increased urinary exosome OPG in ADPKD patients and the presence of OPG in cells lining the cysts. OPG was also found on the luminal side on non-cystic CKD tubules. In ADPKD cyst formation starts during renal development [65], [66] and progresses continuously in adulthood. PKD protein-positive exosomes have been characterized, [67] showing that some key proteins are shed in membrane particles in the urine. Altered tubular cell proliferation and apoptosis as well as abnormal deposition of extracellular matrix contribute to cystogenesis in PKD [68], [69] [70], [71]. An overall predominance of proliferation leads to the increased numbers of tubular cells that line the cysts. Considering the role of OPG in other systems to counterbalance the apoptotic effects of TRAIL, further investigations may focus on the hypothetical apoptotic-regulatory role of OPG in PKD.

The clinical data should be considered as preliminary and hypothesis-generating, as the study is limited by a small number of patients. However, it clearly shows that urinary exosome-like vesicles OPG can be assessed in the urine of CKD patients and a cohort study in underway in order to address whether urinary exosome-like vesicle OPG provides prognostic information on the progression and outcome of CKD.

In summary, we have characterized for the first time proteins present in human proximal tubular cell exosomes. The description of exosomal OPG is novel and points to a role of tubular cell exosomes in the regulation of cell death or inflammatory processes. In addition, the high presence of extracellular matrix proteins suggests a role of tubular cell exosomes in regulation extracellular matrix deposition or cell-matrix interactions under physiological or disease conditions. Further characterization of the function of exosomal OPG and its biomarker potential should be pursued.

## Supporting Information

Figure S1 **SRM of additional OPG peptides.** In addition to the peptide shown in figure 2.D, three additional peptides and their transitions had been monitored. Each depicted window (including 2 or more chromatograms) shows different transitions (fragments) from the same precursor. The peptides monitored were A) GNATHDNICSGNSESTQK; B) SCPPGFGVVQAGTPER and C) CPPGTYLK.
(TIF)

Figure S2 **Western blot confirmation of the presence in proximal tubular cell derived exosomal-like vesicles of representative proteins found in these vesicles by LC MS/MS proteomics:** Representative Western blot for Fibronectin. Lanes contains 10 µg protein obtained from HK2 and 10 ug of tubular cell-derived exosome-like vesicles (ELV).
(TIF)

Figure S3 **Western blot confirmation of the presence in proximal tubular cell derived exosomal-like vesicles of representative proteins found in these vesicles by LC MS/MS proteomics.** Representative Western blot for TGFβ-ih3 and C3. Five micrograms (5 µg) of proximal tubular cell exosome-like vesicles protein were loaded in each lane.
(TIF)

Figure S4 **OPG in human urinary exosomes.** Representative Western blot. 5 µg urinary exosome-like vesicles protein were loaded. DN: Diabetic Nephropathy; GN: Glomerulo Nephritis; ADPKD: autosomal dominant polycystic kidney disease; CAKUT: Congenital anomalies of the kidney and urinary tract.
(TIF)

Table S1 **Proteins identified in tubular epithelial cell-derived exosomes by SDS-PAGE and LC-MS/MS analysis.** The sequences presented in Italic grey are repeated sequences. Con: Confidence; %Cov: Coverage Percentage. Prec MW: precursor Molecular Weight; Theor MW: Theoretical Molecular Weight.
(XLS)
