# Supplementary figures and images for: Osteoprotegerin in Exosome-Like Vesicles from Human Cultured Tubular Cells and Urine
Source: PLoS One. 2013 Aug 23;8(8):e72387. doi: 10.1371/journal.pone.0072387 (PMC3751949; doi:10.1371/journal.pone.0072387)

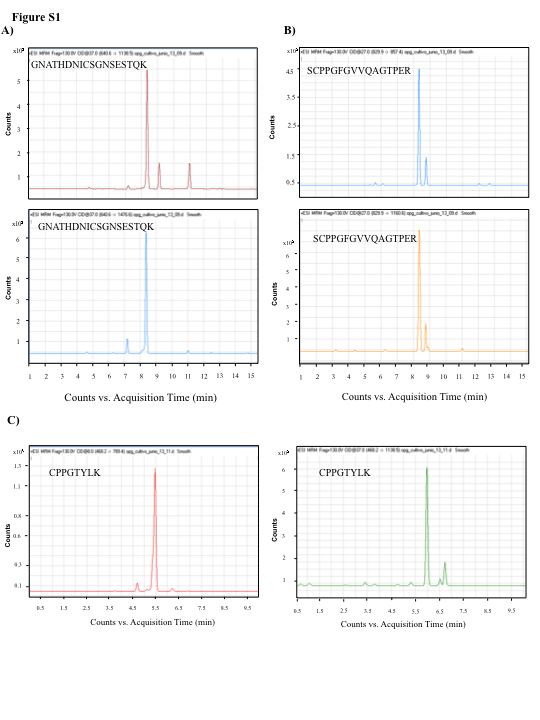

Supplement: Figure S1 — SRM of additional OPG peptides. In addition to the peptide shown in figure 2.D, three additional peptides and their transitions had been monitored. Each depicted window (including 2 or more chromatograms) shows different transitions (fragments) from the same precursor. The peptides monitored were A) GNATHDNICSGNSESTQK; B) SCPPGFGVVQAGTPER and C) CPPGTYLK. (TIF) [file pone.0072387.s001.tif]
